# Osseointegrated Transcutaneous Device for Amputees: A Pilot Large Animal Model

**DOI:** 10.1155/2018/4625967

**Published:** 2018-09-13

**Authors:** Brian T. Grisez, Andrew E. Hanselman, Karim W. Boukhemis, Trapper A. J. Lalli, Brock A. Lindsey

**Affiliations:** Department of Orthopaedics, West Virginia University, PO Box 9196, Morgantown, WV 26506-9196, USA

## Abstract

Traditional above-the-knee amputation prosthetics utilize a stump-socket interface that is well-known for skin/socket problems, sitting difficulty, disuse osteopenia, and increased work of ambulation. As a result, we evaluated a novel osseointegrated transcutaneous implant in a large animal. The implant was designed to promote osseointegration at the bone-implant interface and minimize complications. As proof of concept, four Dorset sheep underwent a two-stage surgery for forelimb placement of an osseointegrated transcutaneous implant utilizing Compress® technology (Biomet, Inc., Warsaw, IN). Two sheep received a long anchor plug (90 mm long x 9 mm in diameter) and two received a short anchor plug (46 mm long x 9 mm in diameter). Sixteen weeks after the initial surgery, the operative limbs, along with the attached implant, underwent radiographic and histological analysis for osseointegration. Periprosthetic fractures occurred in the two animals that received the longer internal prosthesis; one healed with splinting and the other animal underwent a second surgical procedure to advance the amputation site more proximal. No fractures occurred in the shorter internal prosthesis group. There was no histological evidence of infection and none of the transcutaneous adapters failed. Bone-implant osseointegration was demonstrated in two of three limbs that underwent histological analysis. This unique implant demonstrated osseointegration without transcutaneous adapter failure, all while displaying minimal infection risk from the outside environment. Although it involved short-term follow-up in a limited number of animals, this pilot study provides a platform for further investigation into the valid concept of using Compress® technology as an endo-exo device.

## 1. Introduction

Above-the-knee amputations have traditionally utilized a stump-socket interface for prosthetic attachment and ambulation. Unfortunately, this type of prosthetic design is associated with numerous problems. Skin changes (i.e., ulcerations, cysts, and contact dermatitis), increased energy expenditure during ambulation, disuse osteopenia, and sitting difficulty are the substantial issues [1, 2]. These factors, in turn, may lead to limited activity levels and decreased autonomy by amputees [3].

Osseointegrated transcutaneous femoral prosthetics were developed in the 1990s to address these problems [4]. Based on the work of Per-Ingvar Brånemark, who coined the term “osseointegration” with the development of dental implants, the basic prosthetic design consists of an internal intramedullary component, a transcutaneous component, and a detachable external prosthesis [5]. In comparison with stump-socket fixation, these devices have been shown to improve limb control and sensory perception, decrease oxygen consumption with ambulation, improve sitting comfort, and allow more efficient prosthetic donning and doffing [6]. Unfortunately, the transcutaneous nature and design of these implants predispose the amputee to infection, bone fracture, bone loss due to stress shielding, and implant failure [7, 8].

Currently, there are only a few osseointegrated transcutaneous prosthetic systems in use for humans and none of these systems are approved for general use in the United States. The current implant designs, although successful in their own ways, have substantial room for improvement, specifically in regard to the success of the osseointegration, the balance between the amount of bone stock required for implantation and removal of the implant, and the sturdiness of the transcutaneous component. As a result, we believe that large animal models are the appropriate first step in evaluation of a novel implant system utilizing Compress® technology (Biomet Inc., Warsaw, IN). In 2003, the Food and Drug Administration (FDA) approved the Compress® technology for use in limb-salvage procedures; this device provides a constant compressive load across the bone-implant interface. This design may act as a seal for the intramedullary canal, limiting ascending infection from the outside, while allowing for shorter working femoral length and preserving more bone stock if removal is necessary, all while avoiding stress shielding completely [9].

The overall goal of our study was to evaluate a unique osseointegrated transcutaneous implant utilizing Compress® technology and test it using a large animal model. Specifically, we wanted to use this pilot study as a proof-of-concept for acquisition of data that would help us further this research. The specific aims of this study include displaying the ability to surgically implant this novel design, evaluating the bone implant/interface by histology showing potential for complete osseointegration, minimizing infection risk, and obtaining robustness of the transcutaneous adapter without failure.

## 2. Materials and Methods

### 2.1. Implant Design

**Figure 1:**
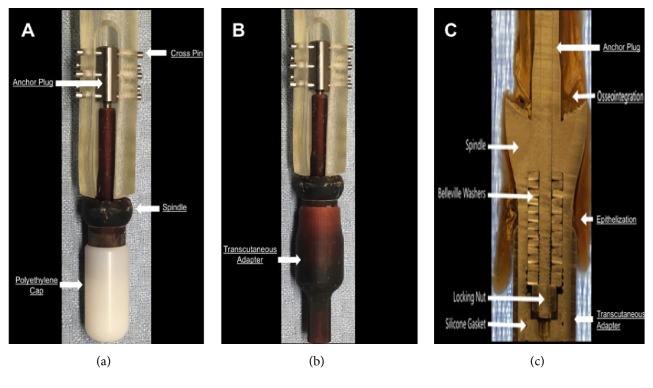
(a) During the first procedure, the spindle and anchor plug are implanted. A cap is placed over the spindle and the soft tissues are closed to allow for provisional healing. (b) The cap is removed at the second surgery and the taper fit transcutaneous adapter is applied. (c) A cross section of the implant demonstrating the compression mechanism. Compression of the spindle against the bone is generated by tightening of the anchor plug locking nut against the Belleville washers. The anchor plug is secured proximally by cross pins (not shown). A silicone gasket fits within the distal end of the spindle to deter bacterial migration proximally into the canal. The taper fit transcutaneous adapter is attached over the distal aspect of the spindle.

A novel intraosseous transcutaneous prosthesis was developed utilizing Compress® technology (Figure 1). It consisted of a proximal intramedullary anchor plug equipped with transverse, bicortical pins to provide rotational stability. The anchor plug was continuous with a Compress® titanium spindle with plasma sprayed titanium porous surface at the bone interface that provided approximately 600 pounds of force (*lbf*) across the bone-implant interface, in order to promote osseointegration. Compression was accomplished through the use of Belleville washers and a locking nut within the prosthesis itself. The internal mechanism of the spindle also contained a silicone gasket to make the seal from the internal prosthesis and medullary canal air and water tight. The spindle/anchor plug device allowed connection to a transcutaneous adapter. This sisal polished cobalt chrome adapter communicated with the outside environment to allow attachment of different external prostheses. Based on the results of prior studies [10, 11], the spindle and transcutaneous adapter were constructed to be smooth and highly polished, in an attempt to promote epithelialization of the skin-implant interface and limit abscess formation creating a stable stoma at the skin-implant interface.

### 2.2. Animal Surgery

**Figure 2:**
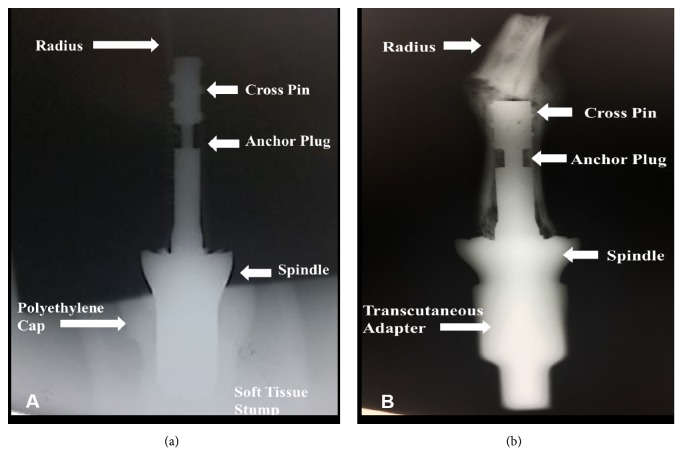
Radiographic images of the staged implant procedure. (a) Implant of spindle components with polyethylene cap and primary wound closure (b) Removal of polyethylene cap which is replaced with a tapered-fit transcutaneous adapter.

Institutional Animal Care and Use Committee* (IACUC*) approval was obtained prior to study initiation. Four three-year-old, skeletally mature, female Dorset sheep (*Archer Farms, Inc., Darlington, MD*) (*average weight = 120 lbs*) underwent a two-stage implant procedure. The first stage consisted of surgical amputation of the forelimb at the distal radius, placement of the Compress® internal prosthesis, polyethylene cap, and closure of the soft-tissue envelope over a polyethylene spacer with an extraction hole distally for removal of the spacer percutaneously (Figures 1 and 2). Six weeks later, a second surgical procedure consisted of externalization of the coupling device through the formation of a transcutaneous portal, removal of the polyethylene spacer, and attachment of the transcutaneous prosthesis (Figures 1 and 2). The forelimb of the sheep was chosen due to its vertical alignment, compared to the hindlimb, thus more closely mimicking the forces experienced by the human femur during stance and movement. Two sheep (*1A and 1B*) received a longer anchor plug (*90 mm*) and two sheep (*2A and 2B*) received a shorter anchor plug (*46 mm*). This change was made because the size and bow of the forelimb contributed to the fractures experienced in the first group. Preoperative and intraoperative anesthesia consisted of xylazine (*0.02-0.2 mg/kg, IV*), ketamine (*7mg/kg, IV*), diazepam* (0.3 mg/kg, IV*), and isoflurane (*5% induction and 1.5-4% maintenance, inhalation*). Preoperative analgesia consisted of lidocaine (*2%, 8-10mL, SQ*) and bupivacaine (*up to 15 mL, SQ*). Postoperative analgesia consisted of fentanyl (*50 micrograms/hr, transdermal*) immediately postoperatively and morphine (*up to 10 mg daily, IM*) and butorphanol (*up to 20 mg, SQ or IV*). Other drugs included excenel* (2.2 mg/kg, SQ*) preoperatively and for three days postoperatively, vitamin B complex (*5mL, SQ*) for four days postoperatively, omeprazole (*1.0 mg/kg, PO*) for seven days postoperatively, probiotics (*1.0 mg/kg, PO*) for three days postoperatively, and kaopectate (*4oz, PO*) for three days postoperatively.

### 2.3. Postoperative Animal Care

During the six-week period between the first and second procedures, the animals were casted and limitation of weightbearing was attempted to limit rotational stress on the device until osseointegration began to occur at the bone spindle interface. However, nearly all animals used the experimental forelimb almost immediately. After the second surgical procedure, the animals were allowed to bear weight as tolerated. Throughout the duration of the study, animals were housed at a local research farm equipped to maintain large animals. Daily care was provided by staff trained in this particular species, with veterinarian care provided as needed. Daily assessment from the start of the study included ambulation ability, use of the prosthesis, bone fracture, and implant failure. Clinical assessment for infection included presence of fever, warmth or swelling of the limb, draining of purulent material from the wound after the first surgery and observation of frank pus and necrotic tissue at the time of the second surgery. Ten weeks after the second stage, or sixteen weeks from the first stage, the sheep were euthanized (*Pentobarbital/Euthasol/Fatal-Plus, 100 mg/kg, IV*) and their operative limbs were harvested.

### 2.4. Limb Analysis

Plain film radiographic images of the harvested limbs with the retained internal prostheses were performed immediately after euthanasia. The limbs with implanted prosthesis were soaked in 10% neutral buffered formalin for 72 hours, then wrapped in formalin-dampened gauze, and sent to the Histomorphometry and Molecular Analysis Core at University of Alabama at Birmingham for histological processing. Here, they were embedded in polymethyl methacrylate (*PMMA*) and cut into four quadrants along their longitudinal and transverse axes in a 90°-90° fashion using an EXAKT diamond band saw. One approximately 100 um section was then prepared from each quadrant using the EXAKT grinding system and stained with methylene blue/basic fuchsin stain (*hematoxylin and eosin-like stain*). They were specifically evaluated for infection* (assessing for inflammation at the interface*), osseointegration at the bone-spindle interface (*defined as >50% surface area of the bone with contact to the spindle*), and epithelialization at the skin-implant interface.

## 3. Results (Table 1)

**Figure 3:**
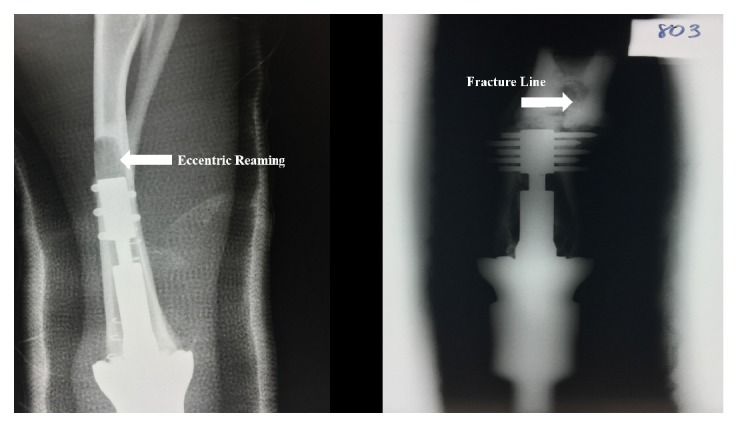
Two animals sustained fractures of their radius after the first surgery. It was presumed that eccentric reaming (left) due to the bow of the radius created a stress riser and resultant spiral type fracture (right)

**Figure 4:**
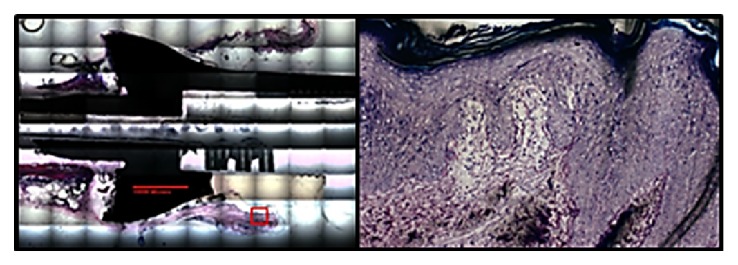
This figure shows the transcutaneous adaption of the skin epithelialization at the implant-skin interface (Sheep 1A).

Sheep 1A received the longer anchor plug (*90 mm*). Seventeen days after the first stage surgical procedure, the animal sustained a spiral type fracture proximal to the implant (Figure 3). Although casted after the initial surgery, the animal was full weight bearing immediately after surgery and did not show any signs of splinting or lameness of the limb prior to the fracture. Three days later, a second surgical amputation was performed moving the internal prosthesis more proximal toward the shoulder (*humerus*) using the same prosthesis because of a custom design. An autoclave was not available at the time of the surgery and the implant could not be sterilized. Six weeks later, the second stage procedure was performed to externalize the internal prosthesis. At that time, abscess formation was appreciated in the soft tissue surrounding the extramedullary portion of the implant. It appeared to be a deep infection with extraosseous involvement. We continued with the externalization procedure and postoperatively treated the animal with an antibiotic (*enrofloxacin*) for six weeks. Upon conclusion of the study, there was no clinical evidence of infection; no wound culturing was performed at the time of the initial infection or at the completion of the study. Since we deviated from the original research protocol by performing a revision of the first stage, we only performed gross histological analysis of the limb after euthanasia. This analysis demonstrated good evidence of epithelialization along the skin-implant interface with minimal downgrowth (Figure 4). No osseous histological analysis was completed on this animal because of such gross deviation from the protocol and it was felt that any further analysis would potentially skew any observations or results.

**Figure 5:**
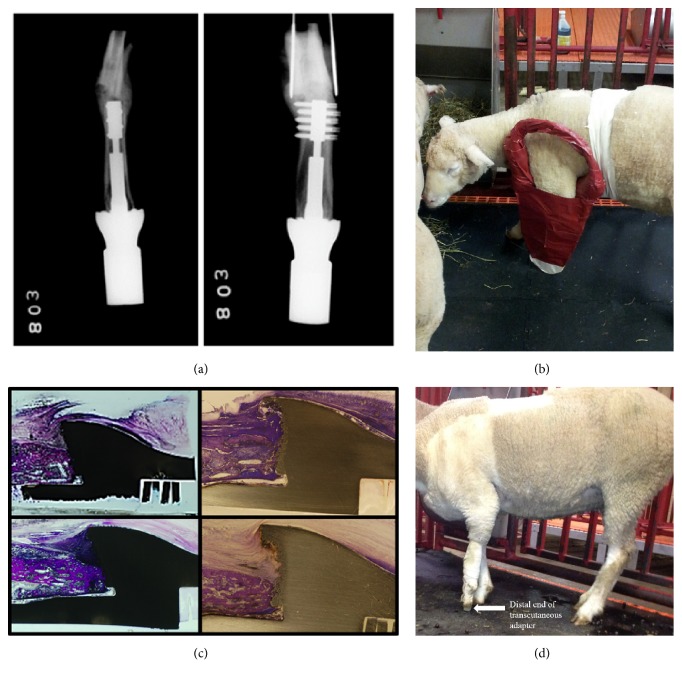
(a) This figure shows radiographs of the implant in Sheep 1B. (b) This figure displays the splinted extremity of the fractured animal, Sheep 1B. (c) This figure demonstrates histological analysis of the osseointegration at all four quadrants in Sheep 1B. (d) This photograph taken after the second surgery demonstrates the animal weight bearing without restriction on the transcutaneous adapter.

Sheep 1B received the longer anchor plug (*90 mm*) (Figure 5(a)). Similar to Sheep 1A, the animal sustained a spiral type fracture proximal to the implant 13 days after the second stage procedure. Unlike Sheep 1A, the implant was retained and the limb was splinted providing rotational control, as well as complete nonweightbearing of the limb (Figure 5(b)). After the limb was harvested, histological analysis revealed bone-implant osseointegration in all four quadrants (Figure 5(c)). Due to the unique surface design of the Compress® spindle base (*a solid structure with macro- and microtexture*), osseointegration is deemed complete with bony onlay at the spindle within the microtexture of the implant. Although this process takes years to complete, histological evidence of bony apposition is consistent with the process of osseointegration occurring. No osseointegration will occur along the anchor plug as this is a smooth surface and acts as a traction bar between the femur and the spindle.

No infection was appreciated clinically or histologically.  Figure 5(d) demonstrates the animal weightbearing without restriction on the transcutaneous adapter after the second surgery.

**Figure 6:**
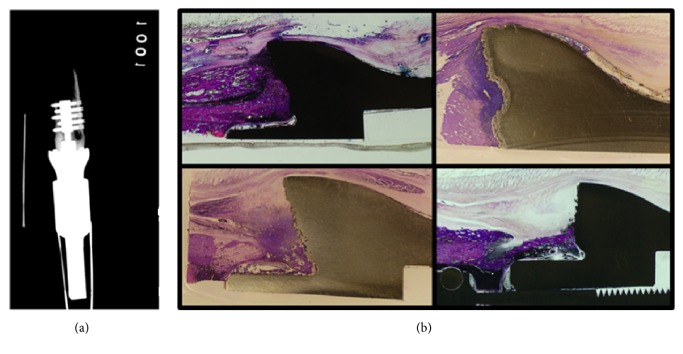
(a) This figure shows radiographs of the implant in Sheep 2A. (b) Histological analysis of Sheep 2A demonstrating osseointegration at all four quadrants.

Sheep 2A received the shorter internal implant (*46 mm*) (Figure 6(a)). No fractures or implant failure occurred; no infection was detected clinically or histologically. Osseointegration was appreciated on histological analysis in all four bone-implant quadrants (Figure 6(b)).

**Figure 7:**
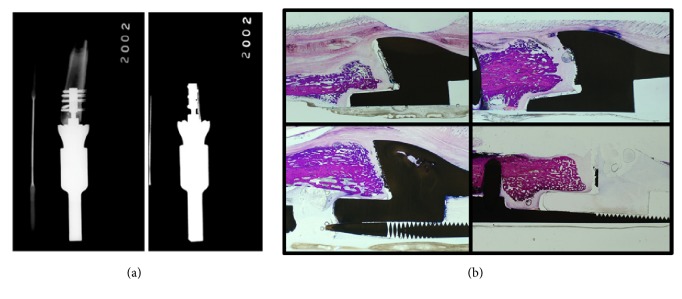
(a) This figure shows radiographs of the implant in Sheep 2B. (b) Histological analysis of Sheep 2B demonstrating tenuous osseointegration in the lower left hand quadrant but only bony encroachment toward the spindle base in the other three quadrants without fulminant osseointegration.

Sheep 2B received the shorter internal implant (*46 mm*) (Figure 7(a)). During the initial operative procedure, a drill bit broke due to surgeon error during implantation of a transverse pin; consequently, we were unable to place a pin at that site. No fractures occurred and no infection was detected clinically or histologically. Osseointegration was not appreciated on histological analysis in three of the four bone-implant quadrants; although bony down-growth toward the spindle interface is apparent (Figure 7(b)).

## 4. Discussion

The aim of our pilot study was to develop a unique osseointegrated transcutaneous implant utilizing Compress® technology and test it in a small number of large animals over a short time period for acquisition of data that would help us develop a platform to continue this research. Specifically, we wanted the implant to demonstrate the potential for complete osseointegration at the bone-implant interface, display limited infection risk from the outside environment, and contain a stout enough transcutaneous adapter to prevent implant failure. Two of three limbs that underwent histology demonstrated bone-implant interface osseointegration in all four quadrants. In one limb that did not demonstrate bony apposition at the implant interface, an intraoperative surgeon error resulted in a reduction in the number of transverse pins that normally provide rotational control and increased stability. Modifying the anchor plug (from 10 mm to 9mm) to decrease the diameter created a ridge for the drill bit to catch causing it to break, which produced a significant amount of torque on the implant.

The two animals with the longer implant sustained fractures. One had a revision wherein the internal prosthesis was placed in the humerus and one healed after application of a splint that restricted weightbearing and provided rotational control. No fractures were observed in the shorter implant group. No evidence of transcutaneous adapter implant failure was observed in any of the animals. We believe the fractures occurred because of a distinct bow in the animal's radius, which did not allow a straight anchor plug of 90 mm to be implanted without some eccentric reaming of the cortical bone (Figure 3). This reaming created a stress riser and as we could not limit the animal's weightbearing, the bone fractured. Future studies could involve a time period of weight bearing restriction postoperatively as part of the established protocol.

No histological evidence of acute infection, presence of acute inflammatory cells (*polymorphonuclear neutrophils*), abscess formations, or bacteria was noted by histology (*Sheep 1B, 2A, and B*). Although some chronic inflammatory cells were noted, this was likely a mild foreign body reaction, which was consistently noted in all the specimens analyzed. One animal demonstrated clinical abscess formation prior to the second stage procedure. However, this complication was likely related to revision surgery since the same internal device was used and unable to be resterilized appropriately, which would logically lead to a higher infection risk. This infection was deep and appeared to be extraosseous in nature. Despite a contaminated environment, there were also no superficial infections. The infection responded well to antibiotic treatment and, even after being off antibiotics, the transcutaneous stoma showed no signs of infection.

Creating a stable stoma around the implant is an important feature in the development of a successful transcutaneous device. Aschoff et al. reported early device designs that utilized a porous transdermal component that was plagued with issues including skin irritation, patient discomfort, hypergranulation, and infection [10, 11]. Pendegrass and colleagues have done extensive investigation into implant-skin interface healing. Common reasons for failure at this interface include marsupialization, avulsion, and infection. Their work has indicated that smooth surfaces improve keratinocyte adhesion and if dermal, not epithelial, attachment occurs and epithelial down growth is prevented [12, 13]. The histology from our implant shows epithelialization of the skin around the transcutaneous adaptor with a stable stoma and minimal down growth at the implant, skin/air/interface. Although this device has maintained the idea of a stable stoma concept with no epidermal integration into the device itself, others (Pitkin et al.) have utilized titanium based pylons that have had some success in feline animal studies which shows some promise [14]. However, no large animal or human studies have been published as of yet.

There are few research groups implanting human devices throughout the world and advancements in this field in the United States have started to gain momentum; most studies use animal models. One study by Drygas et al. evaluated a Siberian husky implanted with bilateral tibial prostheses. The implant comprised a titanium intramedullary stem and a porous tantalum outer sleeve, inserted in a press-fit manner. One limb experienced aseptic loosening at 14 months and required reimplantation; however, the animal was able to walk, trot, and run 26 months postoperatively [15]. Although this study is important, it evaluated the prostheses in tibiae which does not readily translate to the more vertical alignment of the human leg. We chose the sheep model because of similarities to humans in both body weight and bone remodeling capabilities [16, 17]. As stated earlier, the forelimb of the sheep was chosen due to its vertical alignment and its ability to more closely mimic the forces experienced by the human femur [18].

Currently, the greatest contribution to development of an osseointegrated implant for amputees in the US has come from the University of Utah and the Veterans' Affairs Salt Lake City Health Care System. To date, this group has completed numerous studies involving animal models, including sheep, to evaluate implant design. Unlike the osseointegrated compressive design used in our study, their implant consists of a press-fit ribbed titanium stem with a porous coated subcutaneous collar. They have demonstrated good histologic and radiographic evidence of osseointegration as well as decreased infection risk from the outside environment [19–22]. They are currently working toward human trials.

Work underway overseas and at the previously stated US institution has been promising but compressive osseointegrative technology has the potential to achieve superior results. The Compress® system has been FDA approved since 2003 for use in limb-salvage procedures. It has several advantages making it an appealing design choice in the development of new osseointegrated transcutaneous prostheses. First, the technology is based upon Wolff's law in which a compressive force is applied to the bone-implant interface resulting in bone formation and osseointegration [23]. Not only does this design eliminate stress shielding from the implant, but also it seals the intramedullary canal to limit the spread of ascending medullary infection from the outside environment. Over the past few years, the longevity of these implants has been evaluated in orthopaedic literature. A recent study by Monument et al. evaluated 18 patients treated with compressive osseointegration of either the proximal or distal femur with a minimum of five years of follow-up. They demonstrated implant survivorship of 88% when evaluating for aseptic revision and 67% implant survivorship when including revision for oncologic failure, infection, arthrofibrosis, and mechanical failure [24]. A study by Healey et al. showed similar results; they retrospectively reviewed 82 patients treated with the Compress® Knee Arthroplasty for distal femoral replacements. They demonstrated an 85% implant survival rate at 5 years and 80% survival rate at 10 years. Further, of the failed implants, 62% (8/13) were the result of failures at the bone/implant interface [25]. In both studies, most implant failures were observed early in the postoperative period, with little change in outcome after the first two years. The other important advantage of these implants is the minimal amount of bone stock required for both implantation and removal. A study by Tyler et al. evaluated 11 patients who underwent removal of a compressive osseointegrated device from either the proximal or distal femur over an 11-year period. Intraoperative measurements revealed an average bone loss of 8 mm with removal of the implant [9]. Not only do these implants require minimal bone stock for implantation, but also they provide the surgeon with future options if revision surgery is required. The final advantage is the transcutaneous component of this device, which is more robust; in our small series, we had no implant failures. Some may consider the placement of a Compress® device in patients with poor bone quality, as often seen in transfemoral amputees, to be a risk factor for periprosthetic fracture; however, this has not been supported in the literature.[26, 27].

This study has several limitations. First, the number of animals included in the study is small (*N=4*). It is difficult to ascertain statistically significant conclusions based on these numbers; however, the ultimate goal of our study was to obtain data demonstrating the feasibility of this concept. Based on the data shown, this concept as a transdermal osseointegrated prosthesis is promising. Second, the follow-up for this study would be considered short-term at 16 weeks. However, these initial data allow us to feel positive about many of the short-term concerns such as acute infection or implant failure. Third, difficulty maintaining lack of weight bearing was another limitation not initially anticipated. All animals immediately ambulated after the initial amputation and implantation of the spindle. Although the cross pins through the anchor plug do provide some rotational control, the device is designed for limited weight bearing until bony apposition develops along the spindle. Further, this immediate weight bearing likely contributed to the fractures seen in two of the animals. This was successfully addressed with the developed splinting technique. Finally, the last limitation was the use of both long and short anchor plugs in a small study; however, there is not published data that compares one to the other. Although the shorter anchor plug has been commercially available for quite some time without any massive clinical failures, one possible advantage to the use of the shorter plug is in the setting of very little residual femur; this device can still be used with as little as approximately 5 cm of bone. This could potentially take someone who physically acts more like a hip disarticulation, since the residual limb cannot support a prosthesis, to a functional extremity. However, the longer plug may be seen as stronger, since the longer anchor plug creates less cantilevering leading to a more equally distributed force across the end of the bone. Future human trials would allow for more clinically relevant patient-specific outcomes with minimal risk to the patient and with potential profound advantages.

Osseointegrated transcutaneous prosthetic devices for human use are not widely available in the United States and the current implants being used in other countries have several features that could be improved. Taking these limitations into consideration, we felt that the best way to address these issues, such as osseointegration of the bone-implant interface, the amount of bone stock required for implantation/removal, and the sturdiness of the external prosthesis, could be addressed with an implant utilizing Compress® technology. Our proof-of-concept study, using a large animal model, demonstrated osseointegration is possible at the bone-implant interface and no occurrences of transcutaneous adapter implant failures in a worst-case scenario. Although our numbers were small and the results were based on a short time period, the goal of our pilot study was to obtain initial data as a feasibility study to demonstrate that this concept has merit.

Subsequent to this animal study being performed, a case series has been conducted on humans with this device, with substantial success [28]. These devices have been performed under the custom regulatory process. There were three complications in 13 patients: two fractures that were revised and maintained their revised prosthesis and one taper mismatch which was corrected. No infections were noted in this small study. Our group intends on collaborating with the FDA IDE study soon for further investigation.

## Figures and Tables

**Table 1:** Results overview.

**Sheep**	**Infection**	**Fracture**	**Implant Failure**	**Osseointegration**
**1A**	Yes	Yes	No	n/a
**1B**	No	Yes	No	Yes
**2A**	No	No	No	Yes
**2C**	No	No	No	No

## Data Availability

The data resides in the Department of Orthopaedics at West Virginia University; it is largely in the form of histology slides.

## References

[1] Dudek N. L., Marks M. B., Marshall S. C., Chardon J. P. (2005). Dermatologic conditions associated with use of a lower-extremity prosthesis. *Archives of Physical Medicine and Rehabilitation*.

[2] Waters R. L., Mulroy S. (1999). The energy expenditure of normal and pathologic gait. *Gait & Posture*.

[3] Van De Meent H., Hopman M. T., Frölke J. P. (2013). Walking ability and quality of life in subjects with transfemoral amputation: A comparison of osseointegration with socket prostheses. *Archives of Physical Medicine and Rehabilitation*.

[4] Hagberg K., Brånemark R. (2009). One hundred patients treated with osseointegrated transfemoral amputation prostheses - Rehabilitation perspective. *Journal of Rehabilitation Research and Development*.

[5] Branemark P.-I. (1983). Osseointegration and its experimental background. *The Journal of Prosthetic Dentistry*.

[6] Sullivan J., Uden M., Robinson K. P., Sooriakumaran S. (2003). Rehabilitation of the trans-femoral amputee with an osseointegrated prosthesis: The United Kingdom experience. *Prosthetics and Orthotics International*.

[7] Nebergall A., Bragdon C., Antonellis A., Kärrholm J., Brånemark R., Malchau H. (2012). Stable fixation of an osseointegated implant system for above-the-knee amputees: Titel RSA and radiographic evaluation of migration and bone remodeling in 55 cases. *Acta Orthopaedica*.

[8] Tillander J., Hagberg K., Hagberg L., Brånemark R. (2010). Osseointegrated titanium implants for limb prostheses attachments: Infectious complications. *Clinical Orthopaedics and Related Research*.

[9] Tyler W. K., Healey J. H., Morris C. D., Boland P. J., O'Donnell R. J. (2009). Compress® periprosthetic fractures: Interface stability and ease of revision. *Clinical Orthopaedics and Related Research*.

[10] Aschoff H. H., Clausen A., Hoffmeister T. (2009). The endo-exo femur prosthesis--a new concept of bone-guided, prosthetic rehabilitation following above-knee amputation. *Zeitschrift für Orthopädie und Unfallchirurgie*.

[11] Aschoff H. H., Kennon R. E., Keggi J. M., Rubin L. E. (2010). Transcutaneous, distal femoral, intramedullary attachment for above-the-knee prostheses: An endo-exo device. *The Journal of Bone & Joint Surgery*.

[12] Pendegrass C. J., Goodship A. E., Blunn G. W. (2006). Development of a soft tissue seal around bone-anchored transcutaneous amputation prostheses. *Biomaterials*.

[13] Pendegrass C. J., Gordon D., Middleton C. A., Sun S. N., Blunn G. W. (2008). Sealing the skin barrier around transcutaneous implants. *The Journal of Bone & Joint Surgery (British Volume)*.

[14] Farrell B. J., Prilutsky B. I., Kistenberg R. S., Dalton J. F., Pitkin M. (2014). An animal model to evaluate skin–implant–bone integration and gait with a prosthesis directly attached to the residual limb. *Clinical Biomechanics*.

[15] Drygas K. A., Taylor R., Sidebotham C. G., Hugate R. R., Mcalexander H. (2008). Transcutaneous tibial implants: A surgical procedure for restoring ambulation after amputation of the distal aspect of the tibia in a dog. *Veterinary Surgery*.

[16] Peters C. L., Hines J. L., Bachus K. N., Craig M. A., Bloebaum R. D. (2006). Biological effects of calcium sulfate as a bone graft substitute in ovine metaphyseal defects. *Journal of Biomedical Materials Research Part A*.

[17] Willie B. M., Bloebaum R. D., Bireley W. R., Bachus K. N., Hofmann A. A. (2004). Determining relevance of a weight-bearing ovine model for bone ingrowth assessment. *Journal of Biomedical Materials Research Part A*.

[18] Duda G. N., Eckert-Hübner K., Sokiranski R., Kreutner A., Miller R., Claes L. (1997). Analysis of inter-fragmentary movement as a function of musculoskeletal loading conditions in sheep. *Journal of Biomechanics*.

[19] Chou T. G. R., Petti C. A., Szakacs J., Bloebaum R. D. (2010). Evaluating antimicrobials and implant materials for infection prevention around transcutaneous osseointegrated implants in a rabbit model. *Journal of Biomedical Materials Research Part A*.

[20] Jeyapalina S., Beck J. P., Bachus K. N., Chalayon O., Bloebaum R. D. (2014). Radiographic evaluation of bone adaptation adjacent to percutaneous osseointegrated prostheses in a sheep model. *Clinical Orthopaedics and Related Research*.

[21] Jeyapalina S., Beck J. P., Bloebaum R. D., Bachus K. N. (2014). Progression of bone ingrowth and attachment strength for stability of percutaneous osseointegrated prostheses. *Clinical Orthopaedics and Related Research*.

[22] Shelton T. J., Peter Beck J., Bloebaum R. D., Bachus K. N. (2011). Percutaneous osseointegrated prostheses for amputees: Limb compensation in a 12-month ovine model. *Journal of Biomechanics*.

[23] Kramer M. J., Tanner B. J., Horvai A. E., O'Donnell R. J. (2008). Compressive osseointegration promotes viable bone at the endoprosthetic interface: retrieval study of Compress implants. *International Orthopaedics*.

[24] Monument M. J., Bernthal N. M., Bowles A. J., Jones K. B., Randall R. L. (2014). What are the 5-year survivorship outcomes of compressive endoprosthetic osseointegration fixation of the femur?. *Clinical Orthopaedics and Related Research*.

[25] Healey J. H., Morris C. D., Athanasian E. A., Boland P. J. (2013). Compress knee arthroplasty has 80% 10-year survivorship and novel forms of bone failure. *Clinical Orthopaedics and Related Research*.

[26] Pedtke A. C., Wustrack R. L., Fang A. S., Grimer R. J., O'Donnell R. J. (2012). Aseptic failure: how does the compress implant compare to cemented stems?. *Clinical Orthopaedics and Related Research*.

[27] Bhangu A. A., Kramer M. J., Grimer R. J., O'Donnell R. J. (2006). Early distal femoral endoprosthetic survival: Cemented stems versus the Compress® implant. *International Orthopaedics*.

[28] McGough R. L., Goodman M. A., Randall R. L., Forsberg J. A., Potter B. K., Lindsey B. (2017). The Compress® transcutaneous implant for rehabilitation following limb amputation. *Der Unfallchirurg*.

